# Magnetic resonance imaging of cardiac tumors: experience of a tertiary medical center

**DOI:** 10.1186/1532-429X-15-S1-E104

**Published:** 2013-01-30

**Authors:** Ian C Chang, Helina Kassahun, Uma Valeti

**Affiliations:** 1 Medicine, Cardiovascular Division, University of Minnesota, Minneapolis, MN, USA

## Background

Cardiac tumors are rare, with estimated prevalence of 0.002-0.3% at autopsy. Symptoms and prognosis of cardiac tumors depend on size, location, and intrinsic aggressiveness. Cardiac magnetic resonance (CMR) imaging is non-invasive and offers unsurpassed soft-tissue characterization. In this study, we sought to examine the CMR imaging of various cardiac tumors and identify radiologic features of clinical relevance for diagnosis, prognosis, and treatment of the patients.

## Methods

We identified patients referred to the University of Minnesota in the last 5 years (January, 1, 2007-August 31, 2012) for cardiac masses who received CMR imaging and had final diagnoses either by histopathology or clinical evidence. CMR was performed on 1.5T scanners using standardized protocols. An experienced level 3 physician interpreted the CMR studies. Findings on CMR imaging were characterized and correlated with multimodality studies including echocardiography, computed tomography, and positron emission tomography. We correlated the imaging features with clinicopathology and compared to those reported in the literature. Significant clinical impact was defined as establishing a new diagnosis, affecting the decision on invasive surgical procedures, or causing medication change. Clinical impact was determined from chart review.

## Results

We evaluated 32 patients (aged 22-86 years) presenting with cardiac masses. Diagnoses included primary benign tumor (N=4, 12.5%), primary malignant tumor (N=3, 9.4%), metastasis (N=7, 21.9%), thrombus (N=9, 28.1%), and pseudotumor (N=9, 28.1%). Pseudotumor included pericardial cyst, calcified mitral annulus, atrial septal defect occlusion device, hiatal hernia, abscess, and lipomatous hypertrophy. Imaging features commonly seen in malignant tumors included tissue heterogeneity, large size (> 5 cm), the involvement of more than one chamber and adjacent structures, and the presence of contrast enhancement. In addition, we identified a previously unreported pattern of tumor perfusion and contrast enhancement in aggressive malignant tumors; this enhancement gradient could represent varying degrees of vascularity and tumor necrosis. Tumors and thrombi could be differentiated (77.8%) by thrombus correlates: characteristic locations, adjacent scarred myocardium on delayed enhancement imaging, and lack of contrast enhancement. CMR imaging had a significant clinical impact in 71.9% of patients and established a new diagnosis in 21.9%.

## Conclusions

CMR imaging of cardiac masses often makes significant clinical impact by providing invaluable information facilitating accurate diagnosis, prognosis and management. Besides commonly reported features, an enhancement gradient on CMR could potentially be used to identify an aggressive malignant cardiac tumor. This study further supports CMR imaging as a useful and clinically relevant modality for cardiac tumor evaluation.

## Funding

None to disclose.

**Figure 1:**
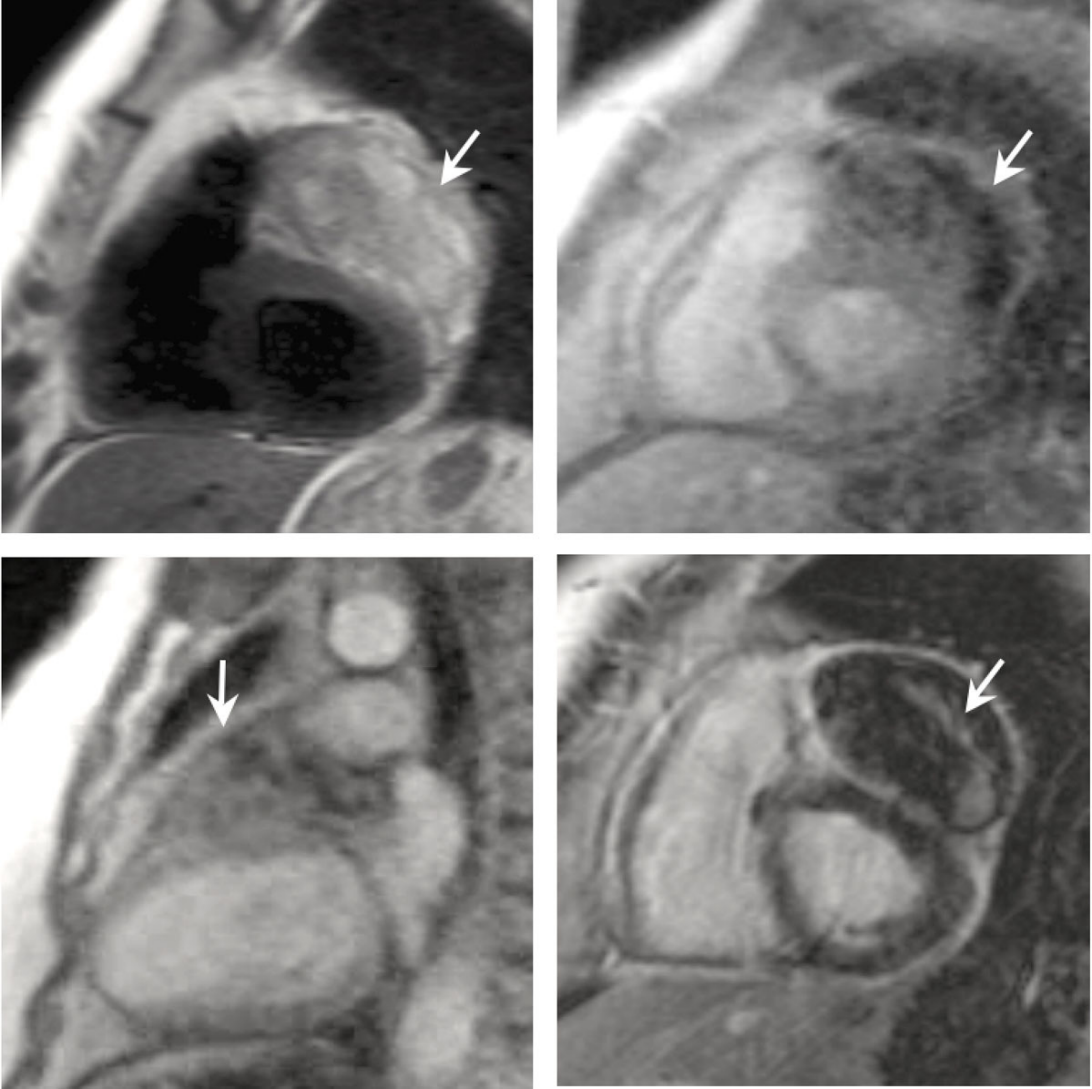
Patient with metastatic undifferentiated sarcoma to the heart (arrow) undergoing CMR imaging. T2-weighted imaging (top left) shows varying signal intensity with more edema in the periphery. Resting perfusion imaging (top right: short axis view, bottom left: long axis view) shows a gradient perfusion with the peripheral portion of the mass having less vascularity and perfusion. Delayed enhancement imaging (bottom right) shows a gradient enhancement suggesting more necrosis along the outer portion of the mass.

**Figure 2:**
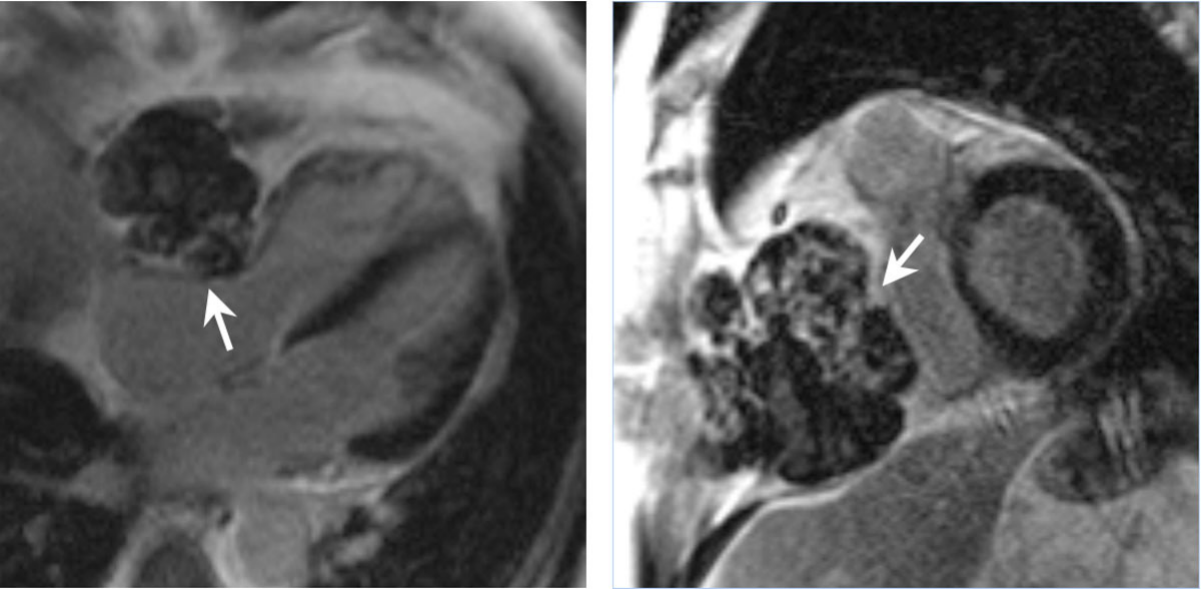
CMR imaging of a patient with metastatic melanoma (arrow) invading the right atrioventricular groove from the mediastinum. Delayed enhancement imaging (left: four chamber view, right: show axis view) shows a gradient enhancement, which suggests more necrosis towards the edges away from the vascular supply of the metastatic mass.

